# Supplementary material for: Trustworthy in silico cell labeling via ensemble-based image translation
Source: Biophys Rep (N Y). 2023 Oct 18;3(4):100133. doi: 10.1016/j.bpr.2023.100133 (PMC10663640; doi:10.1016/j.bpr.2023.100133)
Supplement: Document S1. Figures S1, S2, Table S1, Algorithm S1 [file mmc1.pdf]

Biophysical Reports, Volume *3*

Supplemental information

Trustworthy in silico cell labeling via ensemble-based image translation

Sara Imboden, Xuanqing Liu, Marie C. Payne, Cho-Jui Hsieh, and Neil Y.C. Lin

Trustworthy in silico Cell Labeling via Ensemble-based Image Translation: Supplemental Document

This supplemental document describes the development details of our reported ensemble model. The image perturbation methods and model benmark results are also described.

1. COMPARISON OF UNCERTAINTY ESTIMATION METHODS

To understand how the performance of ensemble-based uncertainty evaluation compares to that of other existing tools, we conducted a systematic comparison of six common methods where three are ensemble-based (i.e. Naive ensemble, BatchEnsemble [1], Snapshot ensemble [2], MC Dropout [3], Stochastic Variational Inference (SVI) [4, 5], and Stochastic Gradient Langevin Dynamics (SGLD) [6, 7]).

A. Parameters for the tested methods

For ensemble-based methods (i.e., naive ensemble, batch ensemble, snapshot ensemble, and our proposed FastEnsemble), we trained six model checkpoints to estimate the uncertainty.

Naive ensemble We trained six models independently with different random seeds. The prediction results were generated by a simple average. The total computational budget is $6B$, where B is the budget to train one model from scratch.

FastEnsemble We first trained a standard checkpoint with budget B, then use $\frac{k_2+k_3}{k_1}B \times 5$ to obtain the rest 5 models. In total, it costed $\frac{k_1+5(k_2+k_3))}{k_1}B$. We chose $k_1 = 200$, $k_2 = k_3 = 6$ for the entire training task.

BatchEnsemble We replicated the batch ensemble code from official repository at https://github.com/google/edward2/blob/main/edward2/tensorflow/layers/convolutional.py#L560, and extended it to support ConvTranspose2d layer. We matched the training budget of our method by increasing the training time proportionally.

MC-Dropout We used the dropout rate equaling to $p = 0.5$. The computational budget is B.

SGLD We first trained the model until convergence (i.e., burn-in phase). At this stage, we did not inject Gaussian noise. During the inference time, we then trained the model for one epoch after each sampling where the learning rate was 1000x smaller than the training stage. No preconditioning technique was applied. We noted that although training budget was only B, the inference budget was much higher than other methods.

SVI We copied the implementation of MFVI from pyro (https://pyro.ai/examples/svi_part_i.html) and a 3rd-party implementation https://github.com/kumar-shridhar/PyTorch-BayesianDNN. The prior follows iid $\mathcal{N}(0, 0.02)$.

B. Quantification of the prediction accuracy

To quantify the performance of uncertainty assessment for individual methods, we used them to evaluate the impact of cell type mismatch and image impurities on prediction accuracy. To do this, we manually labeled the local regions that contain mismatched cells (LNCaP cells) or impurities using bounding boxes (red boxes in Fig. 6a). Here, the cell type mismatch dataset was created by artificially cutting images of LNCaP cells and superimposing them to images of MSCs. Examples of false positive (inside bounding boxes) and false negative (outside bounding boxes) predictions are denoted by yellow arrows in Fig. 6a).

We then defined the pixels inside and outside bounding boxes as positive $\mathcal{S}_1$ and negative $\mathcal{S}_2$ instances, respectively. These pixels were subsequently ranked by the uncertainty values in a descending order. The top-k highest uncertainty pixels $\mathcal{S}_2$ were selected. We then have TP@$k = |\mathcal{S}_1 \cap \mathcal{S}_2|$, Precision@$k$ = TP@k/k, and Recall@k = TP@$k|\mathcal{S}_1|$. Here, Precision@k and Recall@k report the model performance, and TP is the number of true positives.

Using the analyzed result, we then generated a receiver operating characteristic (ROC) curve with TP@k as the y-axis and FP@k as the x-axis (Fig. 6b and 6c) for each method. The ROC curve is a probability curve that reports the true positive rate (TPR) against false positive rate (FPR). To further quantify ROC curves, we computed the area under the curve (AUC) [8], in which

higher AUC values indicate better model performance in distinguishing the positive and negative classes. The results are summarized in Table S1.

We found that the ROC curves of the naive ensemble and our FastEnsemble method exhibit a very similar trend, while we note that the naive ensemble is *sim* 5× slower to train. The runner-up group is the snapshot ensemble and batch ensemble, which are as fast as our FastEnsemble method. Additionally, we found that traditional approximated Bayesian inference methods (i.e., SVI, SGLD and MC-Dropout), did not perform as well as other methods on the tested benchmark. Their suboptimal performance might be due to the approximation being too coarse to make compelling Bayesian inference, suggesting that a more precise Bayesian approximation is required for image translation applications.

2. FAST-ENSEMBLE TRAINING ALGORITHM

Algorithm S1. Algorithm of Fast-ensemble

Initialize: N: number of ensemble models parameterized by w_i; $\ell(\hat{y}, y)$: the loss function; λ: the hyperparameter to be tuned. $k_1 \gg k_2, k_3$: number of iterations for training seeding model, training sub-models and finetuning sub-models.
> Train the seeding model
for all $i \in \{0 \dots k_1 - 1\}$ **do**
    Run one step of optimizer and learning rate scheduler.
Initial model list $\mathcal{M} = \{w_0\}$.
> Train the rest $N - 1$ models
for all $n \in \{1 \dots N - 1\}$ **do**
    for all $i \in \{0 \dots k_2 - 1\}$ **do**
        > Quick training
        One step update to minimize the augmented loss function in Eq. 2.
    for all $i \in \{0 \dots k_3 - 1\}$ **do**
        One step update to minimize the standard loss function in Eq. 1. ▷ Finetuning
    Append to model list $\mathcal{M} = \mathcal{M} + w_n$.

3. ENSEMBLE MODEL OPTIMIZATION

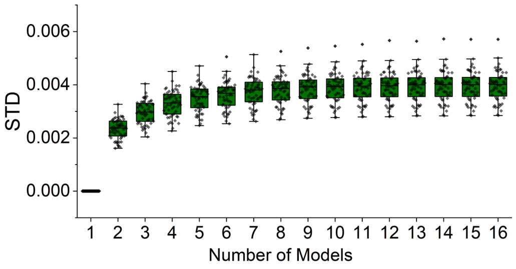

Fig. S1. Ensemble model optimization for model generation and performance. Six models achieved a sufficient STD result with the least computational load. Adding additional models added negligible improvement on statistics.

4. TRANSLATION OF PERTURBED IMAGES

To visualize the increased prediction uncertainty that arises from the image perturbation, we show the uncertainty maps of both perturbed and control (unaltered) images. As shown by the uncertainty maps in Fig. S2, all perturbed input images led to elevated uncertainty values for the cases of overexposure (Fig. S2a), non-uniform illumination (Fig. S2b), and wrong magnification (Fig. S2c). By calculating the target-prediction Pearson correlation coefficient, we confirmed that all imposed image perturbations lowered the AI prediction accuracy (Fig. S2d).

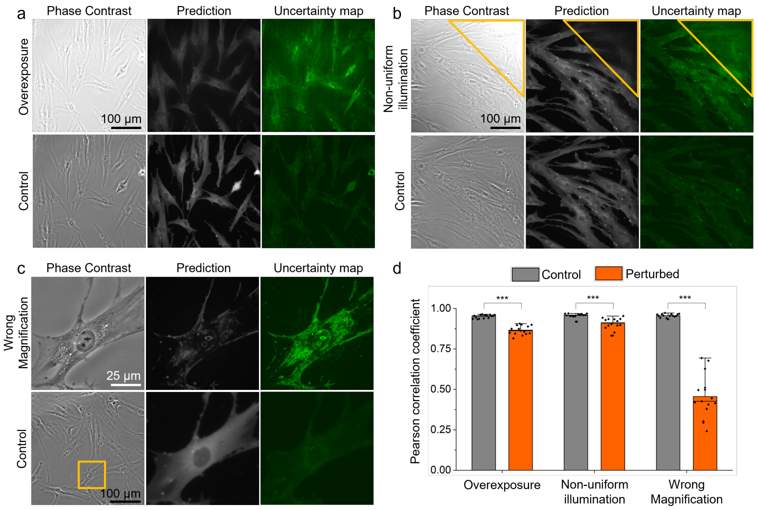

Fig. S2. Comparison of perturbed and corresponding control samples shown in Fig. 4 a-c Example phase contrast and prediction images showing influence of phase contrast perturbation on AI prediction results compared to unperturbed (Control) samples. For all control prediction images, a clear uniform distribution of CD105 can be reported. **d** Mean Pearson correlation coefficient was calculated per FOV for all conditions. We found that a significant drop in Pearson correlation coefficient can be observed for all perturbed FOVs compared to the corresponding control phase contrast image.

5. MODEL COMPARISON AUC RESULTS

Dataset	Naive	MC-Dropout	SGLD	SVI	BatchEnsemble	Snapshot	FastEnsemble
	Area under the ROC curve (AUC, controlling FPR$\leq$ 0.2)						
Image artifacts	**0.112**	0.074	0.099	0.050	0.098	0.002	0.108
Cell type mismatch	0.082	0.035	0.021	0.023	0.059	0.001	**0.090**
	Mean Pearson correlation						
20% Confluency	**0.974**	0.933	0.894	0.887	0.968	0.960	0.971
50% Confluency	**0.952**	0.909	0.853	0.849	0.947	0.939	0.950
80% Confluency	**0.925**	0.869	0.803	0.807	0.919	0.909	0.923
100% Confluency	**0.869**	0.803	0.756	0.762	0.865	0.853	0.865

Table S1. Experimental results in image generation benchmark. For clarity, the highest value is denoted in **bold**, second highest in red, and third highest in blue. Naive Ensemble performs best for all tested tasks except for one (cell type mismatch), confirming the superiority of ensemble methods over Bayesian inference methods in AI image translation tasks.

REFERENCES

1. Y. Wen, D. Tran, and J. Ba, "Batchensemble: an alternative approach to efficient ensemble and lifelong learning," arXiv preprint arXiv:2002.06715 (2020).
2. G. Huang, Y. Li, G. Pleiss, Z. Liu, J. E. Hopcroft, and K. Q. Weinberger, "Snapshot ensembles: Train 1, get m for free," arXiv preprint arXiv:1704.00109 (2017).
3. Y. Gal and Z. Ghahramani, "Dropout as a bayesian approximation: Representing model uncertainty in deep learning," in *international conference on machine learning*, (PMLR, 2016), pp. 1050–1059.
4. M. J. Wainwright and M. I. Jordan, "Introduction to variational methods for graphical models," Foundations Trends Mach. Learn. **1**, 1–103 (2008).
5. D. M. Blei, A. Kucukelbir, and J. D. McAuliffe, "Variational inference: A review for statisticians," J. Am. statistical Assoc. **112**, 859–877 (2017).
6. M. Welling and Y. W. Teh, "Bayesian learning via stochastic gradient langevin dynamics," in *Proceedings of the 28th international conference on machine learning (ICML-11)*, (Citeseer, 2011), pp. 681–688.
7. C. Li, C. Chen, D. Carlson, and L. Carin, "Preconditioned stochastic gradient langevin dynamics for deep neural networks," in *Thirtieth AAAI Conference on Artificial Intelligence*, (2016).
8. C. X. Ling, J. Huang, and H. Zhang, "Auc: A better measure than accuracy in comparing learning algorithms," in *Canadian Conference on AI*, (2003).
