# Supplementary material for: Trustworthy in silico cell labeling via ensemble-based image translation
Source: Biophys Rep (N Y). 2023 Oct 18;3(4):100133. doi: 10.1016/j.bpr.2023.100133 (PMC10663640; doi:10.1016/j.bpr.2023.100133)
Supplement: Document S2. Article plus supporting material [file mmc2.pdf]

Biophysical Reports

ARTICLE

Trustworthy in silico cell labeling via ensemble-based image translation

Sara Imboden,[1] Xuanqing Liu,[2] Marie C. Payne,[1] Cho-Jui Hsieh,[2] and Neil Y. C. Lin[1,3,4,5,6,7,*]

[1]Department of Mechanical and Aerospace Engineering, University of California, Los Angeles, Los Angeles, California; [2]Department of Computer Science, University of California, Los Angeles, Los Angeles, California; [3]Department of Bioengineering, University of California, Los Angeles, Los Angeles, California; [4]Institute for Quantitative and Computational Biosciences, University of California, Los Angeles, Los Angeles, California; [5]California NanoSystems Institute, University of California, Los Angeles, Los Angeles, California; [6]Jonsson Comprehensive Cancer Center, University of California, Los Angeles, Los Angeles, California; and [7]Broad Stem Cell Center, University of California, Los Angeles, Los Angeles, California

ABSTRACT Artificial intelligence (AI) image translation has been a valuable tool for processing image data in biological and medical research. To apply such a tool in mission-critical applications, including drug screening, toxicity study, and clinical diagnostics, it is essential to ensure that the AI prediction is trustworthy. Here, we demonstrate that an ensemble learning method can quantify the uncertainty of AI image translation. We tested the uncertainty evaluation using experimentally acquired images of mesenchymal stromal cells. We find that the ensemble method reports a prediction standard deviation that correlates with the prediction error, estimating the prediction uncertainty. We show that this uncertainty is in agreement with the prediction error and Pearson correlation coefficient. We further show that the ensemble method can detect out-of-distribution input images by reporting increased uncertainty. Altogether, these results suggest that the ensemble-estimated uncertainty can be a useful indicator for identifying erroneous AI image translations.

WHY IT MATTERS Light microscopy has been an essential tool for life sciences and biophysics research. Although many deep-learning models have been introduced to enhance and analyze the microscopy data, these methods have remained underutilized due to the unknown accuracy of AI predictions in practical imaging processing applications. In this work, we demonstrate that a simple ensemble approach that evaluates the standard deviation of independent AI predictions can effectively forecast the prediction accuracy of microscopy data labeling. Using experimental data of mesenchymal stromal cells, we further show that the ensemble method can identify AI prediction errors due to common imaging variations.

INTRODUCTION

Deep learning has demonstrated remarkable promise in enhancing and interpreting biological and medical data that are overwhelmingly complex for traditional parametric approaches (1). Specifically, artificial intelligence (AI) image translation has proved capable of labeling and enhancing image data, in which the deep neural networks (DNNs) decipher the high-dimensional and nonlinear relationship between the target and input images. Such a powerful approach has enabled various in silico analyses of microscopy data, including identification of different cell types in co-culture samples (2), noninvasive labeling of organelles (3), virtual histological staining (4), image resolution enhancement (5), AI-aided medical diagnosis (6), label-free cell sorting (7), and in situ phenotyping of live cells (8,9). These innovative experimental capabilities have paved avenues to addressing interesting open questions in life sciences and biophysical research.

Despite such innovation (2–8,10–19), AI image translation has not been routinely integrated in biology experiments, clinical use, or pharmaceutical applications. A major reason that causes this lack of traction is the unknown accuracy of AI predictions when the ground truth is absent. DNN models are black-box functions with multiple layers of nonlinearities (20), which make the evaluation of prediction confidence challenging (21–23). In the biomedical field, it

Submitted June 16, 2023, and accepted for publication October 16, 2023.

*Correspondence: neillin@g.ucla.edu

Editor: Yoav Shechtman.

https://doi.org/10.1016/j.bpr.2023.100133

© 2023 The Author(s).

This is an open access article under the CC BY license (http://creativecommons.org/licenses/by/4.0/).

is particularly important to ensure the accuracy of AI predictions, as any error could lead to catastrophic misinterpretation such as disease misdiagnosis or false results of drug toxicity or efficacy (24). The inability to know when to trust and when not to trust the AI prediction fundamentally hinders these mission-critical applications of DNNs (25,26).

To address this challenge, various uncertainty estimation methods have been developed in the past decade, mainly for evaluating image classification tasks (27–32); a few of the most popular methods include stochastic gradient Langevin dynamics (SGLD) (33,34), Monte-Carlo dropout (35), stochastic variational inference (SVI) (36,37), and ensemble (e.g., naive, Snapshot (38), and BatchEnsemble (39)). Specifically, the ensemble methods have been developed to report the AI prediction uncertainty by analyzing the prediction distribution of individually trained models. Compared to other popular algorithms, these ensemble-based methods are simple to implement, versatile, and have been shown to perform as well as the Bayesian methods (40–43).

In this work, we adapted the ensemble method to quantify the uncertainty of AI image-to-image translation. To do this, we developed a workflow that converts the standard deviation (Std) of AI predictions into uncertainty. Using actual microscopy data of mesenchymal stromal cells (MSCs), we showed that our ensemble method can effectively capture the AI translation accuracy across multiple molecular markers. More importantly, our method can detect mispredictions that arise from sample mishandling, imaging condition variation, and subtle cellular phenotypic changes, suggesting that the ensemble-based uncertainty evaluation can detect unexpected input (called out-of-distribution (OOD) data). In addition, we developed a FastEnsemble training framework that builds upon the recent findings of the local minimum connectivity in DNNs (44). This training strategy allows us to generate multiple independent ensemble models with a small computational overhead. Experimental results demonstrate that this training framework significantly accelerates the running time without compromising the quality of uncertainty estimation.

MATERIALS AND METHODS

Microscopy image acquisition

Throughout this work, we used experimental microscopy data for testing the uncertainty evaluation method. By imposing tractable perturbations to these microscopy images, we studied how our uncertainty assessment identified AI misprediction.

This work mainly tested microscopy images of MSCs and prostate cancer cells. Specifically, human bone marrow-derived MSCs (ATCC, PCS-500-012) were cultured according to the manufacturer's instruction and standard protocols (45,46). In brief, once the MSCs were thawed, they were seeded into tissue culture flasks at a density of 5000 cells/cm^2 with the culture medium comprising DMEM (Gibco, 1 g/mL glucose, 500 mL), 10% fetal bovine serum (Gibco), and 1% penicillin/streptomycin (Gibco). The MSC culture medium was replaced every 48 h. Similarly to the MSCs, we cultured androgen-sensitive human prostate adenocarcinoma cells (Lymph Node Carcinoma of the Prostate (LNCaP)). LNCaPs were seeded at a density of 10,000 cells/cm^2 and cultured according to ATCC CRL-1740 protocols with medium comprising Gibco RPMI 1640, 10% fetal bovine serum (Gibco), and 1% penicillin/streptomycin (Gibco).

For immunofluorescence, the cells were first washed with PBS+/+; 4% paraformaldehyde (Thermo Fisher Scientific, 28908) in 1× PBS+/+ (Gibco) was subsequently used as the fixative. After ~10 min of incubation, the samples were washed with PBS+/+. To immunostain the sample, the cells were first blocked using a solution consisting of 2% donkey serum (Sigma-Aldrich, D9663-10ML) and 0.5% Triton X-100 (Sigma-Aldrich, T8787-50ML) for 30 min. Each sample was then washed with PBS+/+ twice, and then incubated with the primary staining solution (0.5% BSA, 0.25% Triton X-100, and the primary antibody). The slides were left in the staining solution for 30 min and then washed twice with 1× PBS. After washing, the secondary staining solution (including drops of NucBlue and the secondary antibody (MSC) or BODIPY (LNCaP)) was added for 30 min. Last, the samples were washed twice with PBS+/+ and added to 0.1% Tween 20 (Sigma-Aldrich, P9416-50ML) for long-term storage at 4°C. In addition, a fully prepared and pre-stained mouse kidney section slide was purchased from Invitrogen (F24630).

All samples were imaged using both phase-contrast and fluorescent microscopy (Etaluma LS720, Lumaview 720/600-Series software) with a 20× phase-contrast objective (Olympus, LCACHN 20XIPC).

AI model training image datasets

All AI training datasets consist of paired phase-contrast and fluorescence images of either MSCs, LNCaPs, or kidney tissue section. The image data tested in this work can be mainly categorized into three groups: 1) baseline images that are the raw microscopy data, 2) perturbed images with artifacts that were introduced in a tractable fashion, and 3) OOD images with gradual distribution shifts.

The baseline training images contain pairs of phase-contrast and the corresponding immunofluorescence (IF) images of MSCs. The cells were immunofluorescently stained for a series of surface markers (i.e., CD105, CD29, CD44, CD90, and STRO-1) that are routinely used to define MSC characteristics (47). After image acquisition, quality control was performed where blurry or artifact-containing images were excluded.

To understand if our uncertainty evaluation can be applied in practical cell imaging tasks, we perturbed the baseline training images using Fiji ImageJ (48). We studied the following image perturbations: image impurities, overexposure, nonuniform illumination, and zoomed-in images, which mimicked the effect of using different or compromised microscope settings. Additionally, we investigated the effect of cell type mismatch. These OOD datasets were then used for testing the uncertainty assessment. The training and testing set parameters for each dataset are summarized in Table 1.

To further analyze the performance of our uncertainty assessment, we obtained two sets of training images that have gradual distribution shifts. The first dataset includes images of LNCaP cells that are treated with enzalutamide (Enza) (Selleck Chemicals S1250) for 48 h. Using the untreated sample (control), we trained

TABLE 1 Train and test set parameters for image datasets

Dataset	Training set #	Testing set #
MSC-CD105	616	56
MSC-CD29	279	71
MSC-CD44	337	53
MSC-CD900	616	56
MSC-STRO1	367	58
Image impurities (CD105)	MSC-CD105	488
Overexposure (CD105)	MSC-CD105	15
Nonuniform illumination (CD105)	MSC-CD105	15
Zoom-In/compromised microscopy (CD105)	MSC-CD105	15
Cell type mismatch (CD105, LNCaP)	MSC-CD105	15
Drug-altered phenotype (control)	413	92
Drug-altered phenotype (Enza)	control	96
LNCaP cell density (VSparse)	527	41
LNCaP cell density (Sparse)	VSparse	181
LNCaP cell density (Dense)	VSparse	275
LNCaP cell density (VDense)	VSparse	180
Mouse kidney section (Actin)	300	99

The test set was held separate from the train set for all model testing. All OOD datasets were trained using the MSC-CD105 training set and tested using a separate test set with the corresponding OOD perturbation. For drug-altered phenotype tests, the model was trained using the control condition and tested on either the control or Enza test set. To measure model accuracy on cellular distribution shifts, all LNCaP density datasets were trained on the VSparse training set and tested on the corresponding density test set.

an AI model to predict the fluorescence images of BODIPY (lipid droplets in LNCaP cells) from the phase-contrast images. This model was then applied to the dataset of treated LNCaP cells to evaluate how the drug-altered cell phenotype affects AI predictions. The second dataset comprises images of LNCaP cells with four different cell densities. In these images, the cells exhibit different morphological phenotypes as a result of proliferation. The images were divided into four subsets for evaluation purposes: 20% confluency, 50% confluency, 80% confluency, and 100% confluency. These dataset allow us to systematically study whether our approach is capable of flagging OOD data that have slight distribution shifts.

Comparison of uncertainty evaluation models

To understand how the performance of ensemble-based uncertainty evaluation compares to that of other existing tools, we conducted a systematic comparison of six common methods where three are ensemble based (i.e., naive ensemble, BatchEnsemble (39), Snapshot ensemble (38), MC-Dropout (35), SVI (36,37,49), and SGLD (33,34)). The central features of the tested methods and corresponding parameters used in the comparative study are discussed below.

Naive ensemble

We trained six models independently with different random seeds. The prediction results were generated by a simple average. The total computational budget is 6*B*, where *B* is the budget to train one model from scratch.

FastEnsemble

We first trained a standard checkpoint with budget *B*, then use $\frac{k_2+k_3}{k_1}B\times 5$ to obtain the rest five models. In total, it costed $\frac{k_1+5(k_2+k_3)}{k_1}B$. We chose $k_1 = 200$, $k_2 = k_3 = 6$ for the entire training task.

BatchEnsemble

BatchEnsemble is a method that reduces the computational and memory costs of performing ensemble calculations by optimizing the ensemble weight generation mechanism.(39) We replicated the BatchEnsemble code from official repository at https://github.com/google/edward2/blob/main/edward2/tensorflow/layers/convolutional.py#L560 and extended it to support ConvTranspose2d layer. We matched the training budget of our method by increasing the training time proportionally.

MC-Dropout

The MC-Dropout method is a framework that utilizes dropout training in DNNs as approximate Bayesian inference in deep Gaussian processes.(35). We used the dropout rate equaling to $p = 0.5$. The computational budget is *B*.

SGLD

The SGLD method estimates the prediction uncertainty by adding noise to a standard stochastic gradient optimization algorithm.(33,34). We first trained the model until convergence (i.e., burn-in phase). At this stage, we did not inject Gaussian noise. During the inference time, we then trained the model for one epoch after each sampling where the learning rate was $1000\times$ smaller than the training stage. No preconditioning technique was applied. We noted that, although training budget was only *B*, the inference budget was much higher than other methods.

SVI

The SVI algorithm approximates posterior distributions by conducting stochastic optimization-based variational inference (36,37,49). We utilized the implementation of MFVI from Pyro (https://pyro.ai/examples/svi_part_i.html). The prior follows independent and identically distributed random variables (iid) $\mathcal{N}(0, 0.02)$.

Snapshot ensemble

The Snapshot ensemble algorithm leverages the cyclic learning rate scheduling in stochastic gradient descent to create multiple model snapshots with a single training process (38).

Furthermore, to fairly compare the computational time of these methods, we set the number of epochs to be the same across all methods except for naive ensemble (which is almost five times longer). Although we cannot ensure the wall clock time in each epoch to be the same (SVI and BatchEnsemble tend to be slower due to more complex model architecture), the relative difference in running time is negligible.

Receiver operating characteristic quantification of prediction accuracy

To quantify the performance of uncertainty assessment for individual methods, we used them to evaluate the impact of cell type mismatch and image impurities on prediction accuracy. To do this, we manually labeled the local regions that contain mismatched cells (LNCaP cells) or impurities using bounding boxes (red boxes in Fig. 6 *a*). Here, the cell type mismatch dataset was created by artificially cutting images of LNCaP cells and superimposing them to images of MSCs. Examples of false positives (i.e., the prediction was accurate but flagged by our uncertainty algorithm) and false negatives (i.e., the prediction was inaccurate but

not flagged by our uncertainty algorithm) are denoted by yellow arrows in Fig. 6 *a*.

We then defined the pixels inside bounding boxes as positive instances $\mathcal{S}_1$. All pixels (both inside and outside bounding boxes) were subsequently ranked by the uncertainty values in a descending order. The top-*k* highest uncertainty pixels were then used to define $\mathcal{S}_2$ instances. We then have TP@$k = |\mathcal{S}_1 \cap \mathcal{S}_2|$, Precision@$k$ = TP@k/k, and Recall@k = TP@$k|\mathcal{S}_1|$. Here, Precision@k and Recall@k report the model performance, and *TP* is the number of true positives.

Using the analyzed result, we then generated a receiver operating characteristic (ROC) curve with TP @k as the y axis and FP @k as the x axis (Fig. 6 *b* and *c*) for each method. The ROC curve is a probability curve that reports the true-positive rate against false-positive rate. To further quantify ROC curves, we computed the area under the curve (AUC) (50), in which higher AUC values indicate better model performance in distinguishing the positive and negative classes. The results are summarized in Table S1.

We found that the ROC curves of the naive ensemble and our FastEnsemble method exhibit a very similar trend, whereas we note that the naive ensemble is *sim* 5 × slower to train. The runner-up group is the Snapshot ensemble and BatchEnsemble, which are as fast as our FastEnsemble method. Additionally, we found that traditional approximated Bayesian inference methods (i.e., SVI, SGLD, and MC-Dropout), did not perform as well as other methods on the tested benchmark. Their suboptimal performance might be due to the approximation being too coarse to make compelling Bayesian inference, suggesting that a more precise Bayesian approximation is required for image translation applications.

FastEnsemble

Despite the success of naive ensemble approaches, the naive ensemble method requires independent training for individual models, which could be time consuming for image-to-image training tasks. We propose a simple but effective FastEnsemble method to reduce the training time while maintaining the prediction accuracy. Our approach builds upon the recent findings in mode connectivity of local minimum (44), in which different local minima in the neural network training objective were found to be connected by a "low-loss valley." Therefore, it is possible to traverse from one local minimum to another through a path with small training loss. Starting from the first local minimum w_1, we propose an algorithm to traverse to another local minimum w_2 through this low-loss valley to avoid retraining the model from scratch. Specifically, assume $w_1, \ldots, w_m$ are the current models, to get w_{m+1}, we initialize the model from w_m and solve the following training objective:

$$w_{m+1} = \operatorname{argmin}_w \frac{1}{n} \sum_{i=1}^{n} m(f(x_i; w), y_i) - \frac{\lambda}{m} \sum_{j=1}^{m} \|w - w_m\|1, \quad (1)$$

where the second term in Eq. 1 promotes the diversity of the solutions. The characterization of our FastEnsemble method performance is presented can be found in Table S1. Detailed description of the training algorithm can be found in Algorithm S1.

RESULTS

Ensemble methods for AI prediction uncertainty

The goal of uncertainty estimation is to measure the confidence of the AI model prediction. Previously, uncertainty estimation has been mainly discussed in the context of multi-label classification problems (51–54), where the output of the model, denoted by $f(x; w)$, is a single label. Here, we discuss the implementation of ensemble-based algorithms for uncertainty evaluation in image-to-image translation tasks.

We employed the standard U-Net architecture (55) for our proposed image prediction method. Here, we chose Unet-256 configuration, with channel multiplier (number of filters in the generator = 64) and batch normalization. Dropout is disabled except in the MC-Dropout comparison model. In the model, we assume $f(\cdot; w)$ denotes the neural network parameterized by w. Training a neural network is equivalent to finding the parameters to fit the observed data pairs, which can be written as $\operatorname{argmin}_w \frac{1}{n} \sum_{i=1}^{n} m(f(x_i; w), y_i)$, where $m(\cdot, \cdot)$ is the loss function measuring the discrepancy between the ground truth output and model's prediction and $\{(x_i, y_i)\}_{i=1}^{n}$ are training data. We used stochastic gradient descent as the optimizer for solving the training objective. After training, the model translates each test image x into $f(x; w)$. However, when x is an OOD image, it will still output $f(x; w)$ with suboptimal quality, leading to the importance of uncertainty estimation. For image-to-image translation tasks, we can obtain the uncertainty of individual pixel values from each of the K predictions from each of the K models. One advantage to a spatial uncertainty estimation is to flag regions in predicted images that may be OOD or highly uncertain compared to the rest of the image.

Our uncertainty evaluation model trains $N = 6$ independent CNN models from the same set of training data and runs each model over the test set, which outputs a single-channel, pixel-averaged prediction image (Fig. 1 *a*). Using the difference in pixel values from the generated images, we demonstrate an image map of the Std, with brighter values indicating a higher Std (Std map of Fig. 1 *a*). We found that the ensemble of six independent models is sufficient for generating a robust Std map (Fig. S1). By comparing the Std map to the error map (i.e., deviance between target and mean prediction), we observed a noticeable correlation in the pixel intensity distribution, suggesting that the prediction Std may capture the actual translation error.

Next, we quantified the relationship between Std and error to calibrate the uncertainty across the testing images. We first generated a scatter plot of pixel-level intensity to illustrate this Std-error correlation, where the prediction error diverges monotonically with increasing Std as shown in Fig. 1 *c*. This observed divergence of the Std-error relationship indicates that a greater Std value corresponds to a higher chance of observing larger actual errors. Since the diverging

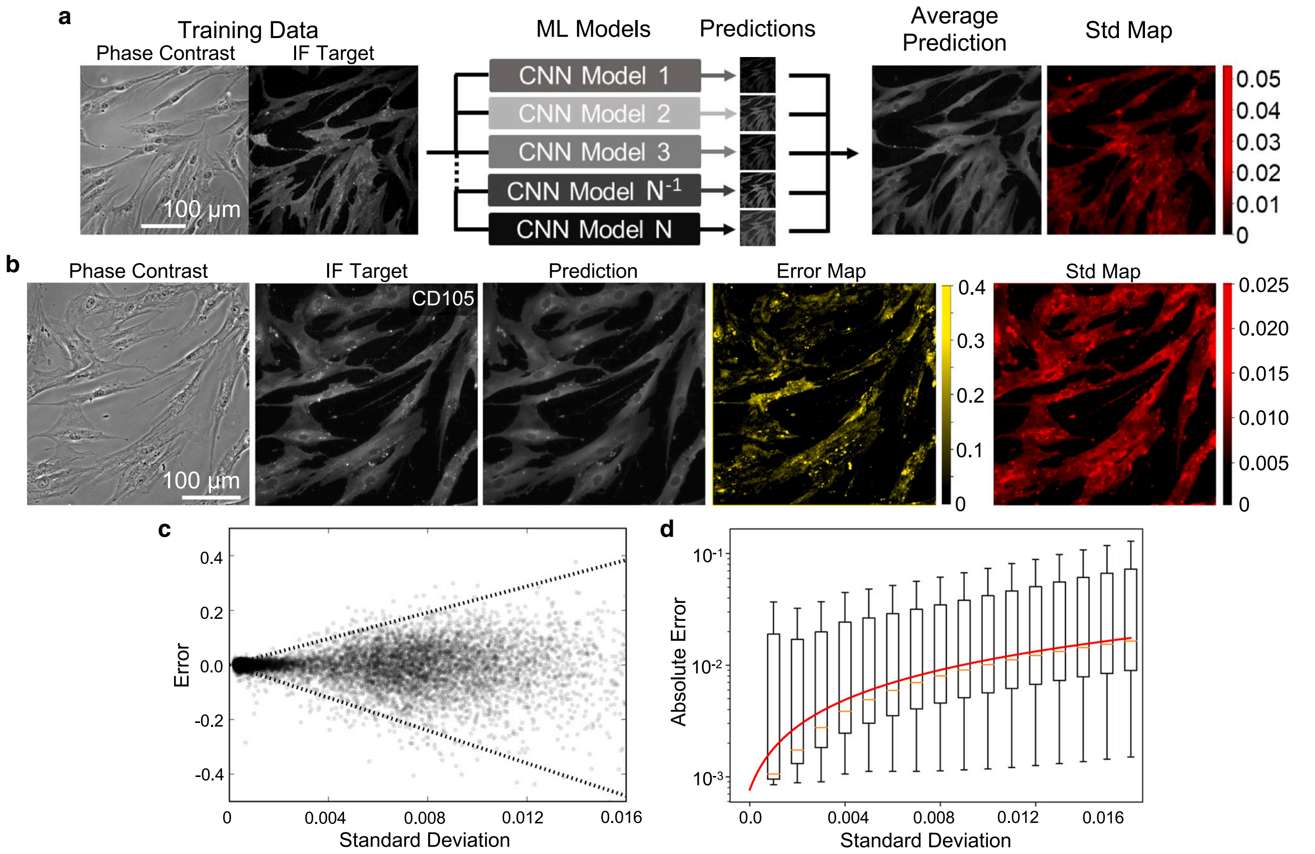

FIGURE 1 Demonstration and characterization of ensemble method and uncertainty map. (*a*) Schematic of the ensemble method, showing independent training of N models that generate an average prediction and corresponding standard deviation (Std) between predictions. (*b*) Left to right: example of a phase-contrast input image, target immunofluorescence image (ground truth), the respective AI prediction, error (difference between prediction and target), and Std map estimated by our ensemble method. (*c*) Scatter plot illustrating the correlation between the pixel-level Std and error divergence (*dashed lines*). (*d*) Boxplot generated by binning the absolute error in Fig. 1 *c*, confirming the correlation between error and Std. Red line denotes a quadratic function fitted to the data $1.318\mathrm{e}-6xStd^2+1.026\mathrm{e}-3xStd+7.662\mathrm{e}-4$. The fitted function is subsequently used for converting Std into uncertainty. Error bars represent standard deviation of the absolute error.

trend of the Std-error relationship cannot be described by an one-to-one function, it is difficult to directly visualize the correlation between error and Std by comparing their maps. Therefore, to further analyze this correlation, we performed equal-width binning of the absolute error and plotted the binned value as a function of Std (Fig. 1 *d*). We then fitted a quadratic polynomial to the mean bin value. Here, we used the quadratic form to describe the Std-error relationship because of its mathematical simplicity and the mildness of the data saturation. In the cases where the data exhibit a more extended saturation plateau, alternative functions, such as exponential saturation, double exponential, and logistic function, can be considered. We also found that the data points of the top 10% of error values are very sparse and can potentially reduce the reliability of fitting. We, therefore, excluded those data points during fitting to ensure that the best fitting curve accurately captures the overall Std-error relationship. The fitted quadratic function (red line in Fig. 1 *d*) was subsequently utilized to determine the pixel-wise uncertainty from the Std. This calibration procedure was repeated for all molecular markers and imaging conditions throughout all experiments.

Ensemble-based uncertainty correlates with AI prediction inaccuracy

To understand whether our uncertainty calculation provides a robust and consistent assessment of the AI prediction accuracy, we performed the uncertainty quantification for five different MSC markers and nucleus staining. For each marker, we averaged the pixel-level uncertainty and absolute error values over individual fields of view (FOVs) and plotted these results in Fig. 2 *a*. We found a positive correlation (Pearson correlation coefficient ~0.83) between our calculated uncertainty and absolute error across all six markers. In addition, the dataset that was contaminated with

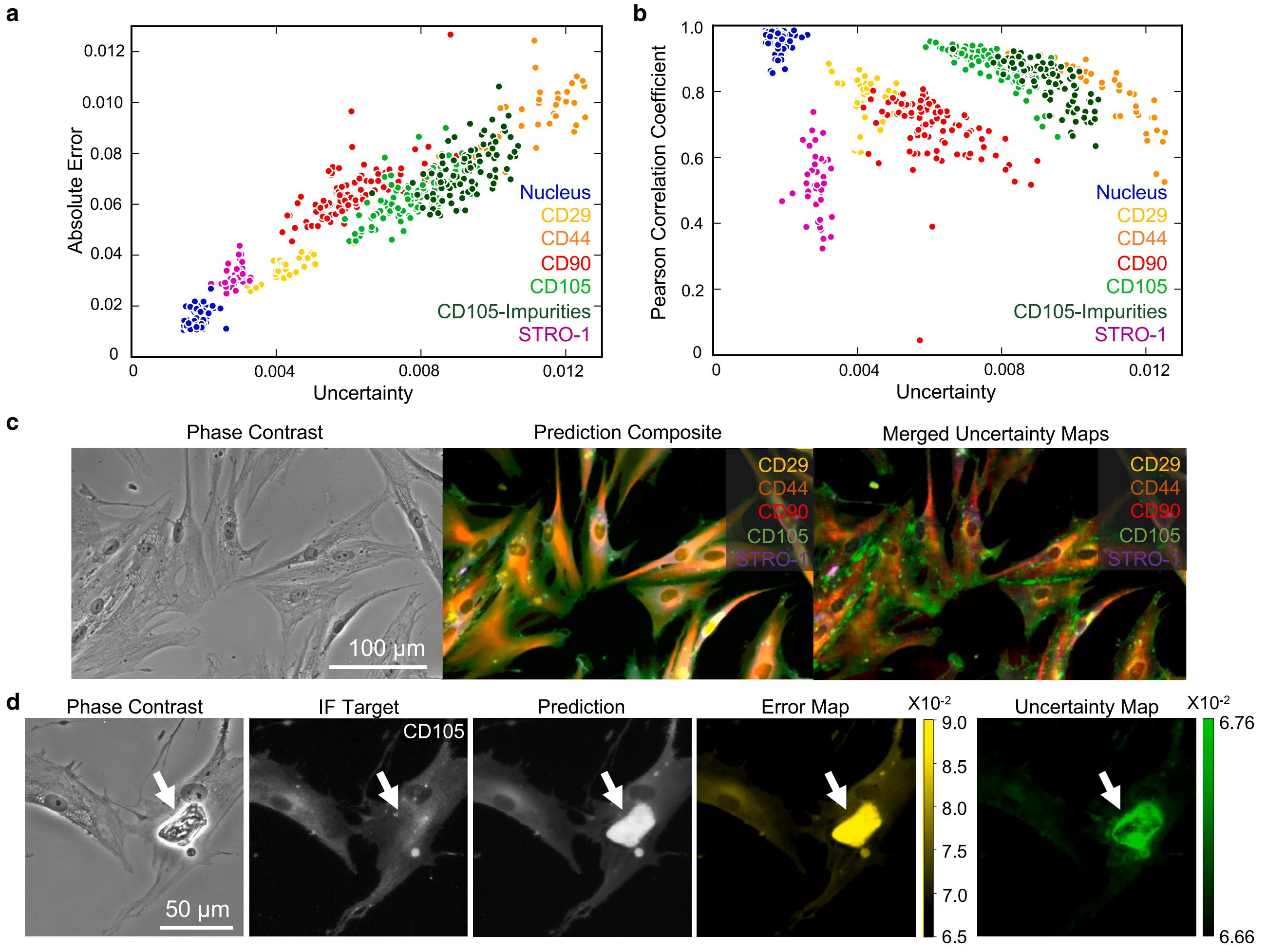

FIGURE 2 Ensemble-estimated uncertainty predicts image translation error across various MSC markers. (*a*) Positive correlation between uncertainty and absolute error that are averaged over the field of view (FOV) for all five tested MSC markers and nucleus staining. In addition, datasets that contain imaging artifacts (*dark green*, CD105-Impurities) also exhibit a similar correlation. (*b*) Uncertainty negatively correlates to Pearson correlation coefficient (r_s) for all tested models. (*c*) Independently trained AI predictions and uncertainty assessments can be combined into an image composite reporting prediction performance for individual markers. (*d*) Ensemble-based uncertainty effectively detects noncellular (*white arrow*) impurities in the image.

imaging artifacts (CD105-Impurities) exhibited a similar trend. This finding supports approximating the error-calibrated Std as uncertainty.

Next, we computed the pixel-pixel Pearson correlation coefficient, r_s, between the target and prediction, in which a higher r_s value indicates a more accurate prediction. By comparing the r_s with mean uncertainty for each FOV in Fig. 2 *b*, we observed a negative correlation between them for all tested markers. Since the definitions of Pearson correlation and uncertainty are strictly independent, we did not anticipate a universal trend across different markers. Our observed anticorrelation between r_s and uncertainty further validated our uncertainty quantification approach. Also, such a finding suggests that our uncertainty assessment is effective for all tested markers. Therefore, an advantage of our uncertainty evaluation method is that it allows us to simultaneously assess the AI prediction performance and combine different markers into one image for an integrative assessment and visualization. An example five-marker composite image is shown in Fig. 2 *c*.

One application of our uncertainty evaluation is to identify noncellular artifacts in the microscopy data. Such impurities may corrupt the analysis statistics and cloud interpretation of AI predictions. The impurities commonly found in microscopy include precipitated crystals in the staining buffer, air bubbles due to pipetting errors, substrate scratches, and bacterial substances. In this work, we tested precipitated crystals as a demonstration. Specifically, we first used impurity-free data (baseline training set) for training the models, and then deployed the trained DNNs for translating phase-contrast images that contain impurities into

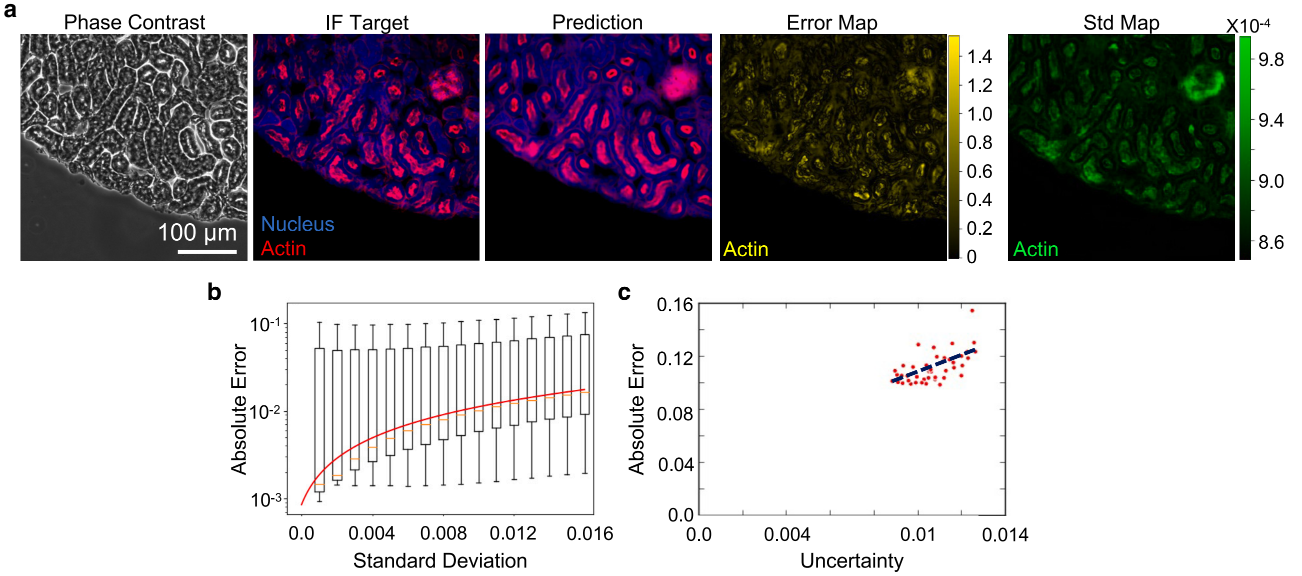

FIGURE 3 Uncertainty evaluation in image translation of mouse kidney tissue sections. Ensemble-based uncertainty can also be used to assess the labeling performance in tissue section images. (*a*) Example images of a mouse kidney section. The sample is fluorescently stained for actin and counterstained for nucleus. In this test, we focused on predicting the actin signal distribution, in which the images from left to right are phase contrast, fluorescent target, AI prediction, target-prediction error map, and the uncertainty map generated using our ensemble method, respectively. (*b*) Boxplot generated by binning the pixel-level absolute error of the actin model, validating the correlation between error and Std. Red line denotes the Std-uncertainty conversion function. Error bars represent standard deviation of the absolute error. (*c*) Positive correlation between uncertainty and absolute error averaged over the FOV.

fluorescent images. As shown in Fig. 2 *d*, the artifact (arrow) caused noticeable mispredictions and strong signal in the error map. At the same time, the pixels that are covered by the contaminant also exhibit uncertainty values that are significantly higher than those of other areas. This result suggests that our uncertainty evaluation can accurately flag the local contaminant that should be excluded from further analysis.

Both the image translation and our uncertainty quantification methods can be applied to a wide range of microscopy applications. We demonstrated this versatility by repeating our uncertainty evaluation using images of mouse kidney tissue sections (Fig. 3 *a*) in which the sample was immunostained for actin and nucleus. Following our calibration workflow, we calculated the prediction Std and converted it into uncertainty. Like the MSC markers, we found that the actin signal in the section sample exhibited a clear error-Std correlation (Fig. 3 *b*). We also found that the converted uncertainty moderately correlated with the absolute error with a Pearson correlation coefficient ~0.57 (Fig. 3 *c*). This demonstration suggests that our uncertainty assessment can be implemented in virtual histological staining and other similar applications.

Evaluation of OOD data uncertainty

The assessments of AI prediction accuracy typically rely on the direct comparison between prediction and target images. In many biological experiments, however, the ground truth images are strictly inaccessible and the prediction-target comparison is practically infeasible. In this case, although the DNN may still generate visually convincing results, these AI-predicted images could potentially deviate from the target. When applied in drug screening, toxicity studies, or clinical applications, such misleading results could have severe consequences (56). The results in the previous subsection suggested that our calibrated uncertainty should be able to detect OOD data. To understand if our quantification method can obtain a reliable metric for AI prediction accuracy without access to the ground truth, we conducted systematic tests using a series of perturbed microscopy images. Specifically, we tested six cases of OOD data, in which the testing datasets exhibit different degrees of visual differences from the actual training set: 1) image overexposure, 2) nonuniform illumination, 3) magnification mismatch, and 4) inconsistent cell type and cell morphology changes arising from 5) drug treatment and 6) cell expansion. These scenarios can be mainly categorized into two groups: imaging condition variations (1–3), and sample variations (4–6).

Applying these perturbations to models trained on the CD105 dataset, we showed that our ensemble method can identify test images that are drastically different from the training data by reporting increased

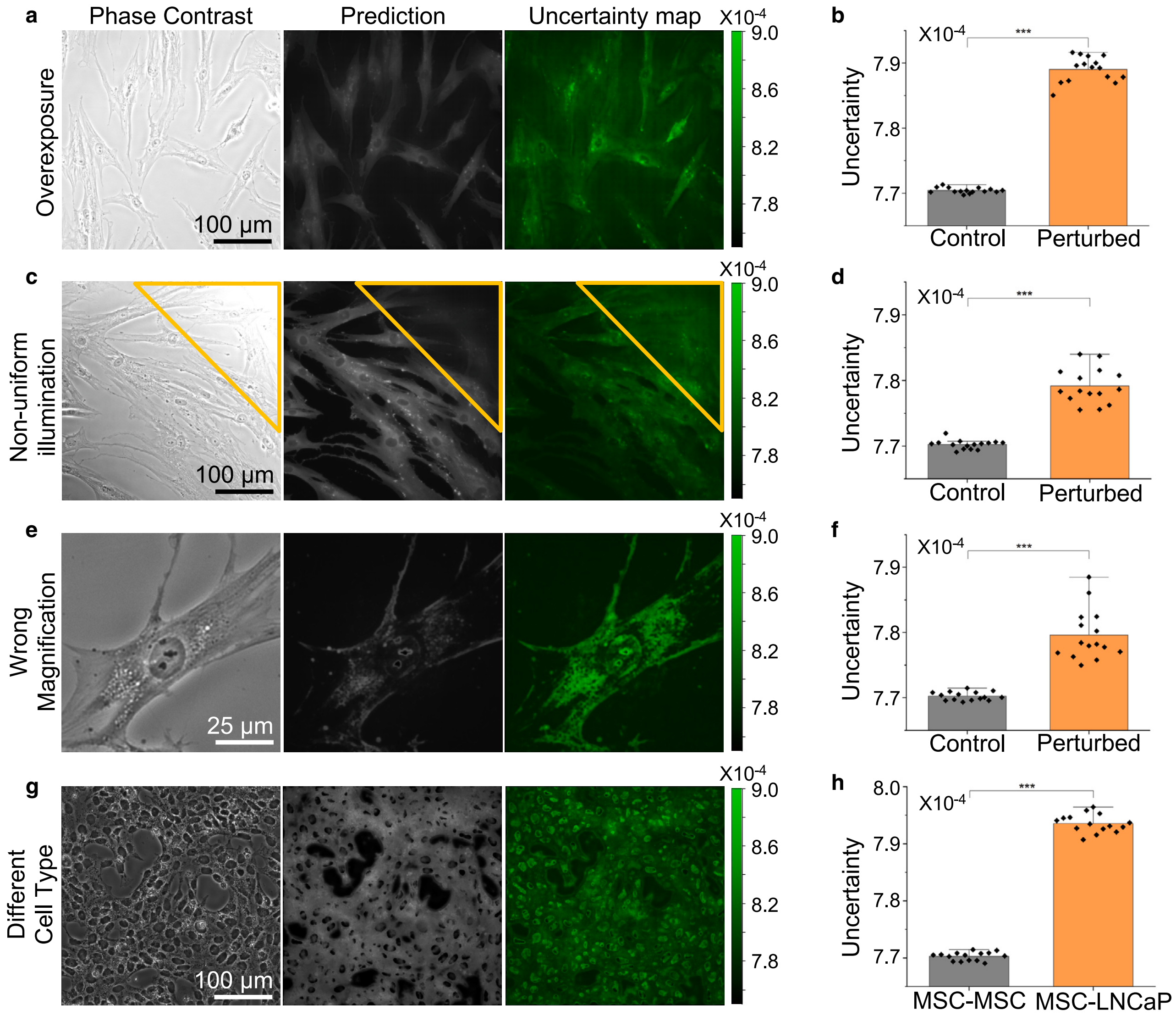

FIGURE 4 Ensemble-based uncertainty flags OOD predictions. The calculated uncertainty successfully identifies OOD image data generated by various types of perturbations, including overexposure (*a* and *b*), nonuniform illumination (*c* and *d*), wrong microscope magnification (*e* and *f*), and different cell type (*g* and *h*). Significant increase in uncertainty values ($n = 15$ FOVs) is observed in all perturbation cases. Note that the y axis limits of (*b*), (*d*), (*f*), and (*h*) were adjusted to highlight the differences between conditions. For all barplots, ***$p < 0.0001$, in which the p values were determined using two-sample Student's *t*-test. Error bars represent standard deviation.

uncertainty values. We selected the CD105 dataset due to the high signal-to-noise ratio of the target and accurate AI predictions. We generated the perturbed images by modifying duplicated test images (i.e., phase-contrast images) using Fiji ImageJ. As shown in Fig. 4 *a*–*f*, we found that all of the introduced image perturbations successfully led to AI prediction errors. We noticed that, although these errors may be detected by trained experts, such mispredictions are subtle and can be easily overlooked. For example, the imaging overexposure caused an overall blurry prediction with faint cell boundaries (Fig. 4 *a*). In the case of nonuniform illumination (Fig. 4 *c*), the overexposed upper right corner (yellow triangle) of the predicted image shows a cloudy and nonlocalized protein distribution. When the magnification is mismatched, we observed a patchy and fragmented CD105 distribution across the cell (Fig. 4 *e*), despite CD105 being a glycoprotein surface marker that should be uniformly expressed throughout the cytoplasm.

Importantly, we found that our uncertainty assessment successfully identified all these subtle errors. First, the uncertainty maps showed elevated intensities for all altered images (Fig. S2), in which the uncertainty assessment correctly highlighted the upper right corner in the nonuniform illumination case. By averaging the pixel-level uncertainty value over the FOV, our method reported a significant increase in

the mean uncertainty compared to the unperturbed data for all tested OOD (bar charts in Fig. 4 *b*, *d*, and *f*). This finding suggests that the uncertainty mean can act as an indicator, labeling predicted images that should be further scrutinized or even excluded. We further studied whether our uncertainty calculation can detect prediction errors due to cell type mismatch. To do so, we applied an AI model that was trained using the MSC data to the images of prostate cancer cells (LNCaP), which should not express CD105. As anticipated, the AI prediction contains substantial errors, which were mostly detected by our uncertainty assessment (Fig. 4 *g* and *h*).

Uncertainty evaluation of data with gradual distribution shifts

An essential application of AI image translation is to provide real-time molecular-based characterizations of cells for pharmacological study. In this application, the AI model predicts the cell characteristics or expression levels of molecular markers that can either evolve during natural cell growth or be altered by drug treatments. Because of cellular dynamics, it is imperative to ensure that the cell behavior is faithfully reported by the AI prediction; however, it has remained difficult to verify the black-box predictions in those tasks (57). In this work, we studied the influence of drug treatment and cell confluency on the AI prediction and tested if our uncertainty estimation is able to detect prediction errors.

To study how the drug-altered cell morphology affects the AI prediction, we cultured prostate cancer cells (LNCaP) and treated them with Enza, which impairs cell growth and alters cell metabolism through androgen receptor inhibition. A specific effect of Enza treatment is the reduction of lipid droplets (i.e., lipogenesis) (58). Using the untreated samples, we first trained an AI model that translates phase-contrast images into fluorescent images of BODIPY that stains the lipid droplets (Fig. 5 *a*). We then applied this model to the test dataset of treated LNCaP cells to obtain BODIPY image predictions (Fig. 5 *b*). Compared to the control data, we confirmed that the LNCaP cell morphology was altered and the BODIPY signal was reduced (IF target) by the Enza treatment. We also found that the AI model that was trained only using the control data cannot fully capture the drug-induced reduction in BODIPY signal (prediction image in Fig. 5 *b*). Such a misprediction can be visualized by the increased intensity in the error map (error map in Fig. 5 *b*). At the same time, we found that the ensemble method reports an elevated level of uncertainty (uncertainty map in Fig. 5 *b*). By averaging the uncertainty value and absolute error over the FOV, Fig. 5 *c* and *d* show that our uncertainty quantification effectively detects the OOD.

To understand how the cell proliferation-induced sample alterations influence the AI prediction, we seeded the LNCaP cells at a relatively low density (20%) and acquired microscopy images until the cells reached 100% confluency (i.e., 100% area coverage). The distribution shift of this dataset mainly arises from phenotypic changes associated with cell density. Like the drug experiment, we trained a model using control data, which are phase-contrast and BODIPY images of 20%-confluency samples (VSparse training set in Table 1). We then applied this model to the datasets of 50% (Sparse), 80% (Dense), and 100% (VDense training set in Table 1) cell confluence (Fig. 5 *e*). We found that the ensemble-predicted uncertainty increases with increasing confluency (Fig. 5 *f*), consistent with the mean target-prediction error calculation (Fig. 5 *g*). This finding further confirms that the cell morphology and density changes directly affect the AI translation accuracy, which can be captured by the ensemble uncertainty calculation.

Ensemble acceleration preserves the uncertainty evaluation accuracy

Although the naive ensemble has been shown to provide am accurate estimate of prediction uncertainty, a major weakness of this method is its computational overhead for building independent models. Therefore, it is critical to investigate new approaches to develop uncertainty estimation methods with low-cost computation. To address this technical hurdle, we developed an acceleration algorithm, FastEnsemble, which searches for independent low-loss optimization paths starting from the initial model (see section "materials and methods"). We found that this directed optimization approach allows us to complete a new model training task with only $3\%–5\%$ additional training time. For example, generating an ensemble of six models using the naive ensemble approach requires a computational time $\sim 6\times$ single-model training time, whereas the FastEnsemble approach requires $\sim 1.2\times$ single-model training time, quintupling the training speed.

Furthermore, such an acceleration preserves the high prediction accuracy achieved by the naive ensemble. We empirically found that this approach can lead to a diverse set of models $w_1, \ldots, w_K$, which gives an uncertainty estimation akin to the naive ensemble method (Fig. 6). To quantitatively assess the performance of the FastEnsemble method, we used bounding boxes to manually label local image regions that comprise inaccurate predictions. These

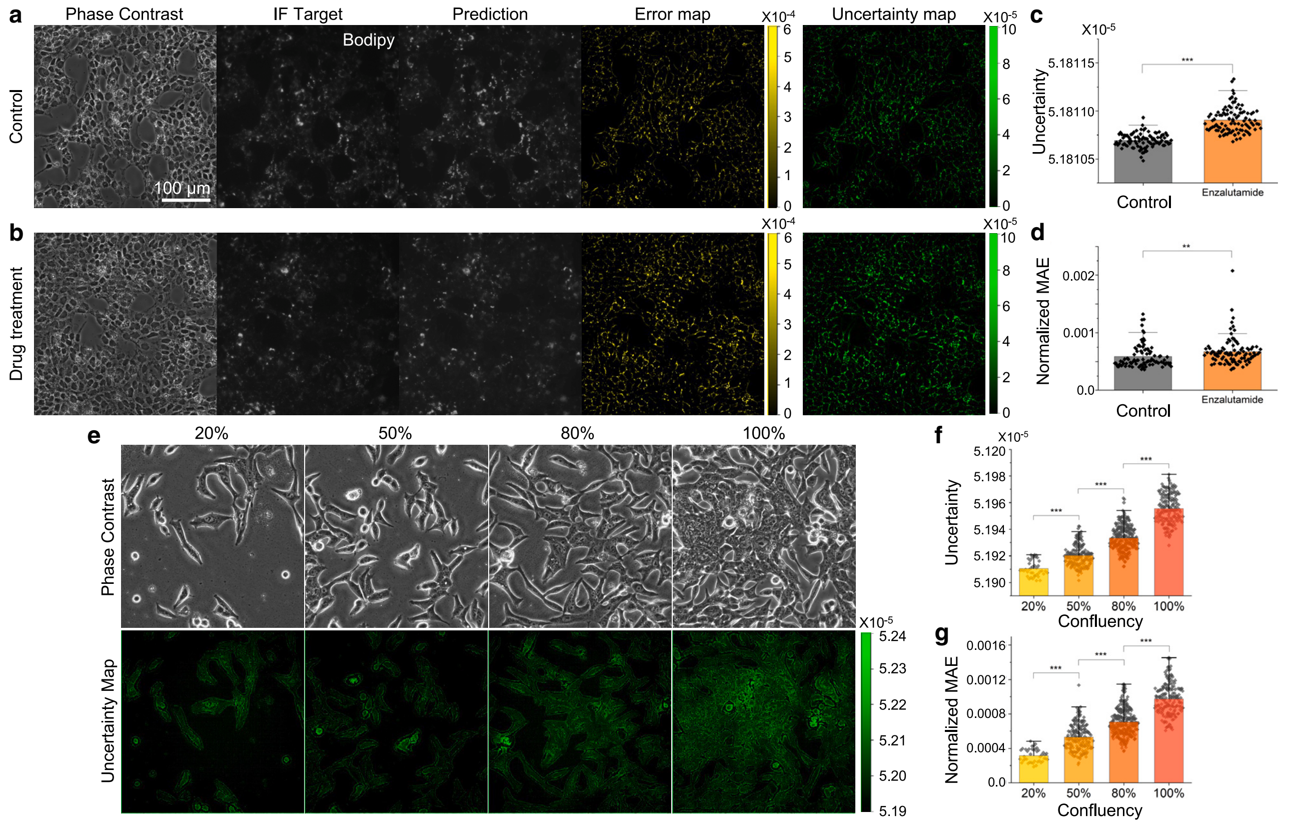

FIGURE 5 Distribution shift-based uncertainty evaluation Our uncertainty estimation is able to detect OOD datasets. (*a*) Example images of the control model, which shows LNCaP cells stained for BODIPY. (*b*) Control model applied to phase-contrast images of LNCaPs that have been treated with a chemo drug called enzalutamide (Enza). Treated samples show slight changes in morphology that lead to significantly higher uncertainty estimation (*c*) in predictions compared to control group. (*d*) Mean absolute error (MAE) calculations verify uncertainty estimation. (*e*) Cell culture expansion leads to confluency (cell density) difference over time, which can affect cell morphology and protein expression profiles. Uncertainty maps show increase in uncertainty estimation with increasing cell confluency. (*f* and *g*) Visual assessment can be confirmed by numeric uncertainty and MAE comparison. Note that the y axis limits of (*c*) and (*f*) were adjusted to highlight the differences between conditions. For all barplots, $^{**}p < 0.001$ and $^{***}p < 0.0001$, in which the p values were determined using two-sample Student's *t*-test. Error bars represent standard deviation.

inaccurate prediction incidents were either artificially introduced (cell type mismatch) or identified using phase-contrast input images (impurity).

We utilized our uncertainty evaluation methods to classify incidents within these labeled image data. Further details of this analysis can be found in section "materials and methods." The uncertainty assessment accuracies of our FastEnsemble and naive ensemble methods were then compared with five previously published methods (i.e., MC-Dropout, SVI, SLGD, BatchEnsemble, and Snapshot ensemble). The main features and corresponding parameters of the tested methods are summarized in the "materials and methods" section. We then analyzed the corresponding ROC curves (see section "materials and methods"), as summarized in Fig. 6 and Table S1. We found negligible performance difference between the FastEnsemble and naive ensemble methods, both of which outperformed the MC-Dropout (blue curve) and SVI (orange) methods. This result demonstrates that we can preserve the performance while reducing computational load. Notably, our FastEnsemble is approximately five times faster than naive ensemble, since each model training is initialized from previous solution, substantially reducing the optimization iterations.

CONCLUSIONS AND DISCUSSION

In this work, we found that ensemble-based deep learning can effectively report the image translation uncertainty, which is correlated with the absolute error and Pearson correlation coefficient. We considered the problem of uncertainty estimation and calibration in image translation under various distribution shifts, in which the batch effects (e.g., cell-cell phenotype variation, batch-to-batch inconsistency, imaging condition differences) are the main reasons

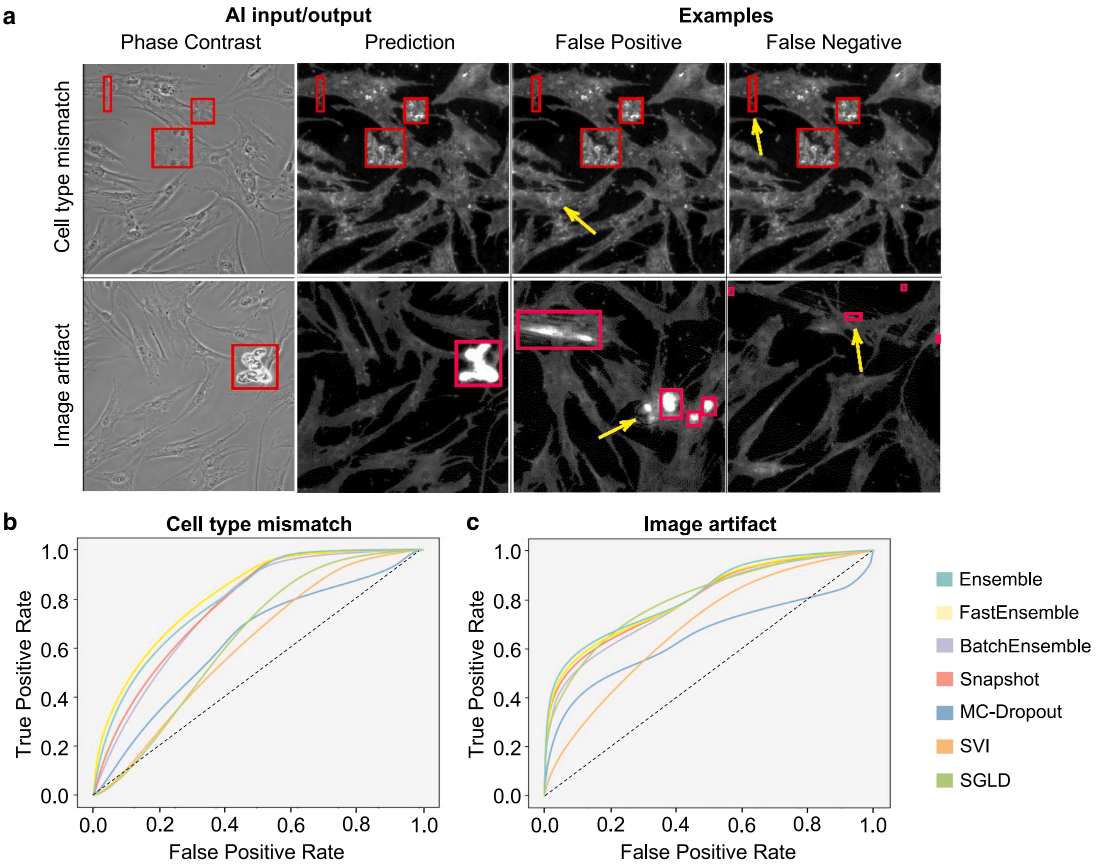

FIGURE 6 Quantification of the prediction accuracy. (*a*) Bounding boxes highlighting OOD area in the test images. Image samples from the cell type mismatch dataset (*top*) and image artifact dataset (*bottom*). Common mispredictions include distorted cell contours (*upper row*) associated with cell type mismatches and speckles (*lower row*) associated with impurities in the experiment sample. The yellow arrows indicate representative false-positive (*third column*) and false-negative (*fourth column*) events. Three different FOVs are shown for the image artifact case (*lower row*), since we could not identify any FOV that contains multiple false-positive and false-negative incidents. (*b* and *c*) Comparison of different ensemble-based uncertainty evaluations and other algorithms. ROC curves showing the relationship between the true-positive rate and false-positive rate for identifying the cell type mismatches (*b*) and image artifacts (*c*). We found that naive ensemble and FastEnsemble methods perform similarly, suggesting that both methods have a comparable sensitivity and specificity to diagnose translation predictions.

responsible for the prediction error associated with different imaging conditions and methods. We further showed that our uncertainty assessment method can be used to forecast translation error that is associated with varying imaging conditions and specimen alterations.

Compared to previous uncertainty studies that have mainly focused on classification tasks, our work demonstrated that ensemble algorithms can be employed to predict image-to-image translation errors, in which experimental microscopy data were directly used for all conducted tests. Furthermore, our FastEnsemble method, which provides a sensitivity and specificity akin to the naive ensemble and other ensemble-based methods (e.g., BatchEnsemble and Snapshot ensemble), presents a valuable alternative approach to reduce computational costs in approximating posterior distribution. Since the ensemble method simply evaluates the statistics of the AI predictions, it can be straightforwardly integrated with various deep-learning models that have been utilized for labeling and processing microscopy data, improving the trustworthiness of these cutting-edge tools (2,3,8,9,59–62).

Overall, our model serves as a foundational step toward robust AI image translation for biomedical applications; however, practical implementation of our reported algorithms would require further development. For example, although the proposed algorithm focuses on pixel-pixel deviations, data-exclusion decisions would require tools to flag highly uncertain features in the microscopy data (63). In addition, although many studies have empirically demonstrated the feasibility of ensemble-based uncertainty evaluation, its underlying working mechanism remains not fully understood. Thus, establishing further theoretical

understanding will enable developments of more reliable uncertainty assessment tools. Last, it is imperative to eventually improve the robustness of AI labeling, where the AI model can still make accurate predictions when encountering OOD images. In robust machine learning, which is a rapidly growing field, studies on distribution shift robustness are specifically related to the microscopy application. Over the past decade, promising advances have been made in developing algorithms to quantify the model robustness (64,65) and training frameworks to optimize the model stability (66–68).

DATA AND CODE AVAILABILITY

Source code and software installation instructions are publicly available at: https://github.com/xuanqing94/BNNBench. All datasets listed in Table 1 can be accessed at the dryad repository (https://datadryad.org/stash/share/sylqXwvnQC Mq6UzlxOS1OsFqPe9DzX1DMtlmKBo603E) except for the MSC-CD105 dataset, which has been previously published and re-analyzed for this study. This dataset can be downloaded from https://ucla.box.com/s/7tbedrg3g6snr528xhnlf9r ivnt8o6q6.

SUPPORTING MATERIAL

Supporting material can be found online at https://doi.org/10.1016/j.bpr.2023.100133.

AUTHOR CONTRIBUTIONS

S.I., X.L., M.P., C.H., and N.L. designed the research. S.I. and X.L. carried out all experiments and model training. S.I., X.L., and M.P. analyzed the data. S.I., X.L., M.P., C.H., and N.L. wrote the article.

ACKNOWLEDGMENTS

We thank Roy Wollman for the fruitful discussion. This work was supported by the UCLA SPORE in Prostate Cancer grant (P50 CA092131), the Eli and Edythe Broad Center of Regenerative Medicine and Stem Cell Research at UCLA and California NanoSystems Institute at UCLA Planning Award grant, the UCLA California NanoSystems Institute and the Noble Family Innovation Fund, the UCLA Hellman Fellowship, the National Science Foundation (NSF CMMI-2029454, CBET-2244760, and DBI 2325121), and the National Institutes of Health NIGMS (1R35GM146735).

DECLARATION OF INTERESTS

The authors declare that they have no known competing financial interests or personal relationships that could have appeared to influence the work reported in this paper.

REFERENCES

1. Choi, R. Y., A. S. Coyner, …, J. P. Campbell. 2020. Introduction to Machine Learning, Neural Networks, and Deep Learning. *Transl. Vis. Sci. Technol.* 9:14.
2. Christiansen, E. M., S. J. Yang, …, S. Finkbeiner. 2018. In silico labeling: predicting fluorescent labels in unlabeled images. *Cell.* 173:792–803.e19.
3. Ounkomol, C., S. Seshamani, …, G. R. Johnson. 2018. Label-free prediction of three-dimensional fluorescence images from transmitted-light microscopy. *Nat. Methods.* 15:917–920.
4. Rivenson, Y., H. Wang, …, A. Ozcan. 2019. Virtual histological staining of unlabelled tissue-autofluorescence images via deep learning. *Nat. Biomed. Eng.* 3:466–477.
5. Wang, H., Y. Rivenson, …, A. Ozcan. 2019. Deep learning enables cross-modality super-resolution in fluorescence microscopy. *Nat. Methods.* 16:103–110.
6. Venkatesan, D., A. Elangovan, …, B. Vellingiri. 2022. Diagnostic and therapeutic approach of artificial intelligence in neuro-oncological diseases. *Biosens. Bioelectron. X.* 11, 100188. https://www.sciencedirect.com/science/article/pii/S2590137022000826.
7. Tang, R., L. Xia, …, Y.-H. Lo. 2023. Low-latency label-free image-activated cell sorting using fast deep learning and AI inferencing. *Biosens. Bioelectron.* 220, 114865. https://www.sciencedirect.com/science/article/pii/S0956566322009058.
8. Imboden, S., X. Liu, …, N. Y. C. Lin. 2021. Investigating heterogeneities of live mesenchymal stromal cells using AI-based label-free imaging. *Sci. Rep.* 11:6728.
9. Weber, L., B. S. Lee, …, N. Y. Lin. 2023. Phenotyping senescent mesenchymal stromal cells using AI image translation. *Current Research in Biotechnology.* 5, 100120.
10. Rivenson, Y., H. Wang, …, A. Ozcan. 2019. Virtual histological staining of unlabelled tissue-autofluorescence images via deep learning. *Nat. Biomed. Eng.* 3:466–477. https://doi.org/10.1038/s41551-019-0362-y.
11. Belthangady, C., and L. A. Royer. 2019. Applications, promises, and pitfalls of deep learning for fluorescence image reconstruction. *Nat. Methods.* 16:1215–1225. https://doi.org/10.1038/s41592-019-0458-z.
12. Ouyang, W., A. Aristov, …, C. Zimmer. 2018. Deep learning massively accelerates super-resolution localization microscopy. *Nat. Biotechnol.* 36:460–468. https://doi.org/10.1038/nbt.4106.
13. Nehme, E., L. E. Weiss, …, Y. Shechtman. 2018. Deep-STORM: super-resolution single-molecule microscopy by deep learning. *Optica.* 5:458–464. http://www.osapublishing.org/optica/abstract.cfm?URI=optica-5-4-458.
14. Moen, E., D. Bannon, …, D. Van Valen. 2019. Deep learning for cellular image analysis. *Nat. Methods.* 16:1233–1246. https://doi.org/10.1038/s41592-019-0403-1.
15. Weigert, M., U. Schmidt, …, E. W. Myers. 2018. Content-aware image restoration: pushing the limits of fluorescence microscopy. *Nat. Methods.* 15:1090–1097. https://doi.org/10.1038/s41592-018-0216-7.
16. Kandel, M. E., Y. R. He, …, G. Popescu. 2020. Phase imaging with computational specificity (PICS) for measuring dry mass changes in sub-cellular compartments. *Nat. Commun.* 11:6256.
17. Narotamo, H., J. Sanches, and M. Silveira. 2019. Segmentation of Cell Nuclei in Fluorescence Microscopy Images Using Deep Learning. Springer-Verlag, pp. 53–64.
18. Chen, Y., Y. Lin, …, J. Huang. 2023. Multi-domain medical image translation generation for lung image classification based on generative adversarial networks. *Comput. Methods Progr. Biomed.* 229, 107200. https://www.sciencedirect.com/science/article/pii/S0169260722005818.
19. Li, Z., J. Jiang, …, W. Chen. 2021. Development of a deep learning-based image quality control system to detect and filter out

ineligible slit-lamp images: A multicenter study. *Comput. Methods Progr. Biomed.* 203, 106048. https://www.sciencedirect.com/science/article/pii/S0169260721001231.

20. Panayides, A. S., A. Amini, …, C. S. Pattichis. 2020. AI in Medical Imaging Informatics: Current Challenges and Future Directions. *IEEE J. Biomed. Health Inform.* 24:1837–1857.
21. Kendall, A., and Y. Gal. 2017. What uncertainties do we need in bayesian deep learning for computer vision?. Preprint at arXiv, arXiv:1703.04977.
22. Hüllermeier, E., and W. Waegeman. 2021. Aleatoric and epistemic uncertainty in machine learning: An introduction to concepts and methods. *Mach. Learn.* 110:457–506.
23. Abdar, M., F. Pourpanah, …, S. Nahavandi. 2021. A review of uncertainty quantification in deep learning: Techniques, applications and challenges. *Inf. Fusion.* 76:243–297.
24. Davenport, T., and R. Kalakota. 2019. The potential for artificial intelligence in healthcare. *Future Healthc. J.* 6:94–98.
25. Rossi, F. 2018. Building Trust in Artificial Intelligence. *J. Int. Aff.* 72:127.
26. Cheng, M., S. Nazarian, and P. Bogdan. 2020. There Is Hope After All: Quantifying Opinion and Trustworthiness in Neural Networks. *Frontiers in Artificial Intelligence.* 3. https://www.frontiersin.org/article/10.3389/frai.2020.00054.
27. Geifman, Y., G. Uziel, and R. El-Yaniv. 2018. Bias-Reduced Uncertainty Estimation for Deep Neural Classifiers. Preprint at arXiv. https://doi.org/10.48550/arXiv.1805.08206.
28. Nado, Z., N. Band, …, G. Jerfel. 2021. Uncertainty Baselines: Benchmarks for Uncertainty & Robustness in Deep Learning. Preprint at arXiv. https://doi.org/10.48550/arXiv.2106.04015.
29. Filos, A., S. Farquhar, …, Y. Gal. 2019. A systematic comparison of bayesian deep learning robustness in diabetic retinopathy tasks. Preprint at arXiv.
30. Menze, B., L. Joskowicz, …, C. Berger. 2020. Quantification of Uncertainties in Biomedical Image Quantification. *Zenodo*. https://doi.org/10.5281/zenodo.3947852.
31. Zimmerer, D., J. Petersen, …, K. Maier-Hein. 2020. Medical Out-of-Distribution Analysis Challenge. *Zenodo*. https://doi.org/10.5281/zenodo.3961376.
32. Gehr, T., M. Mirman, …, M. Vechev. 2018. AI2: Safety and Robustness Certification of Neural Networks with Abstract Interpretation. *In* 2018 IEEE Symposium on Security and Privacy (SP), pp. 3–18.
33. Welling, M., and Y. W. Teh. 2011. Bayesian learning via stochastic gradient Langevin dynamics. *In* Proceedings of the 28th international conference on machine learning (ICML-11) Citeseer, pp. 681–688.
34. Li, C., C. Chen, …, L. Carin. 2016. Preconditioned stochastic gradient Langevin dynamics for deep neural networks. *In* Thirtieth AAAI Conference on Artificial Intelligence.
35. Gal, Y., and Z. Ghahramani. 2016. Dropout as a bayesian approximation: Representing model uncertainty in deep learning international conference on machine learning. *PMLR* 1050–1059.
36. Wainwright, M. J., and M. I. Jordan. 2008. Introduction to variational methods for graphical models. *Foundations and Trends in Machine Learning.* 1:1–103.
37. Blei, D. M., A. Kucukelbir, and J. D. McAuliffe. 2017. Variational inference: A review for statisticians. *J. Am. Stat. Assoc.* 112:859–877.
38. Huang, G., Y. Li, …, K. Q. Weinberger. 2017. Snapshot ensembles: Train 1, get m for free. Preprint at arXiv. https://doi.org/10.48550/arXiv.1704.00109.
39. Wen, Y., D. Tran, and J. Ba. 2020. Batchensemble: an alternative approach to efficient ensemble and lifelong learning. Preprint at arXiv. https://doi.org/10.48550/arXiv.2002.06715.
40. Lakshminarayanan, B., A. Pritzel, and C. Blundell. 2016. Simple and Scalable Predictive Uncertainty Estimation using Deep Ensembles. Preprint at arXiv. https://arxiv.org/abs/1612.01474.
41. Ovadia, Y., E. Fertig, …, J. Snoek. 2019. Can you trust your model's uncertainty? Evaluating predictive uncertainty under dataset shift. Preprint at arXiv. https://doi.org/10.48550/arXiv.1906.02530.
42. Gustafsson, F. K., M. Danelljan, and T. B. Schon. 2020. Evaluating scalable bayesian deep learning methods for robust computer vision. *In* Proceedings of the IEEE/CVF Conference on Computer Vision and Pattern Recognition Workshops, pp. 318–319.
43. Izmailov, P., P. Nicholson, …, A. G. Wilson. 2021. Dangers of Bayesian Model Averaging under Covariate Shift. Preprint at arXiv. https://doi.org/10.48550/arXiv.2106.11905.
44. Garipov, T., P. Izmailov, …, A. G. Wilson. 2018. Loss surfaces, mode connectivity, and fast ensembling of dnns. *In* Proceedings of the 32nd International Conference on Neural Information Processing Systems, pp. 8803–8812.
45. Adamzyk, C., T. Emonds, …, S. Neuss. 2013. Different Culture Media Affect Proliferation, Surface Epitope Expression, and Differentiation of Ovine MSC. *Stem Cell. Int.* 2013, 387324. https://doi.org/10.1155/2013/387324.
46. Hagmann, S., B. Moradi, …, T. Gotterbarm. 2013. Different culture media affect growth characteristics, surface marker distribution and chondrogenic differentiation of human bone marrow-derived mesenchymal stromal cells. *BMC Muscoskel. Disord.* 14:223.
47. Dominici, M., K. Le Blanc, …, E. M. Horwitz. 2006. Minimal criteria for defining multipotent mesenchymal stromal cells. The International Society for Cellular Therapy position statement. *Cytotherapy.* 8:315–317. http://www.sciencedirect.com/science/article/pii/S1465324906708817.
48. Schindelin, J., I. Arganda-Carreras, …, A. Cardona. 2012. Fiji: an open-source platform for biological-image analysis. *Nat. Methods.* 9:676–682. https://doi.org/10.1038/nmeth.2019.
49. Hoffman, M. D., D. M. Blei, …, J. Paisley. 2013. Stochastic variational inference. *J. Mach. Learn. Res.*
50. Ling, C. X., J. Huang, and H. Zhang. 2003. AUC: A Better Measure than Accuracy in Comparing Learning Algorithms. *In* Canadian Conference on AI.
51. Abdar, M., M. Samami, …, S. Nahavandi. 2021. Uncertainty quantification in skin cancer classification using three-way decision-based Bayesian deep learning. *Comput. Biol. Med.* 135, 104418.
52. Carneiro, G., L. Zorron Cheng Tao Pu, …, A. Burt. 2020. Deep learning uncertainty and confidence calibration for the five-class polyp classification from colonoscopy. *Med. Image Anal.* 62, 101653.
53. Mena, J., O. Pujol, and J. Vitrià. 2021. A survey on uncertainty estimation in deep learning classification systems from a bayesian perspective. *ACM Comput. Surv.* 54:1–35.
54. Sensoy, M., L. Kaplan, and M. Kandemir. 2018. Evidential deep learning to quantify classification uncertainty. *Adv. Neural Inf. Process. Syst.* 31
55. Ronneberger, O., P. Fischer, and T. Brox. 2015. U-net: Convolutional networks for biomedical image segmentation. *In* International Conference on Medical image computing and computer-assisted intervention Springer, pp. 234–241.
56. Laine, R. F., I. Arganda-Carreras, …, G. Jacquemet. 2021. Avoiding a replication crisis in deep-learning-based bioimage analysis. *Nat. Methods.* 18:1136–1144.
57. Möckl, L., A. R. Roy, and W. E. Moerner. 2020. Deep learning in single-molecule microscopy: fundamentals, caveats, and recent developments (Invited). *Biomed. Opt Express.* 11:1633–1661. http://opg.optica.org/boe/abstract.cfm?URI=boe-11-3-1633.
58. Cai, C., and S. P. Balk. 2011. Intratumoral androgen biosynthesis in prostate cancer pathogenesis and response to therapy. *Endocr. Relat. Cancer.* 18:R175–R182.

59. Chen, X., M. E. Kandel, …, G. Popescu. 2023. Artificial confocal microscopy for deep label-free imaging. *Nat. Photonics.* 17:250–258.

60. Jo, Y., H. Cho, …, Y. Park. 2021. Label-free multiplexed microtomography of endogenous subcellular dynamics using generalizable deep learning. *Nat. Cell Biol.* 23:1329–1337.

61. Liu, X., B. Li, …, D. Ta. 2023. Virtual Fluorescence Translation for Biological Tissue by Conditional Generative Adversarial Network. *Phenomics.* 3:408–420.

62. Cross-Zamirski, J. O., E. Mouchet, …, Y. Wang. 2022. Label-free prediction of cell painting from brightfield images. *Sci. Rep.* 12, 10001.

63. Angelopoulos, A. N., A. P. Kohli, …, Y. Romano. 2022. Image-to-image regression with distribution-free uncertainty quantification and applications in imaging. *In* International Conference on Machine Learning PMLR, pp. 717–730.

64. Subbaswamy, A., R. Adams, and S. Saria. 2021. Evaluating model robustness and stability to dataset shift. *In* International conference on artificial intelligence and statistics PMLR, pp. 2611–2619.

65. Taori, R., A. Dave, …, L. Schmidt. 2020. Measuring robustness to natural distribution shifts in image classification. *Adv. Neural Inf. Process. Syst.* 33:18583–18599.

66. Krueger, D., E. Caballero, …, A. Courville. 2021. Out-of-distribution generalization via risk extrapolation (rex). *In* International Conference on Machine Learning PMLR, pp. 5815–5826.

67. Subbaswamy, A., and S. Saria. 2020. From development to deployment: dataset shift, causality, and shift-stable models in health AI. *Biostatistics.* 21:345–352.

68. Zhang, X., P. Cui, …, Z. Shen. 2021. Deep stable learning for out-of-distribution generalization. *In* Proceedings of the IEEE/CVF Conference on Computer Vision and Pattern Recognition, pp. 5372–5382.

Biophysical Reports, Volume *3*

Supplemental information

Trustworthy in silico cell labeling via ensemble-based image translation

Sara Imboden, Xuanqing Liu, Marie C. Payne, Cho-Jui Hsieh, and Neil Y.C. Lin

Trustworthy in silico Cell Labeling via Ensemble-based Image Translation: Supplemental Document

This supplemental document describes the development details of our reported ensemble model. The image perturbation methods and model benmark results are also described.

1. COMPARISON OF UNCERTAINTY ESTIMATION METHODS

To understand how the performance of ensemble-based uncertainty evaluation compares to that of other existing tools, we conducted a systematic comparison of six common methods where three are ensemble-based (i.e. Naive ensemble, BatchEnsemble [1], Snapshot ensemble [2], MC Dropout [3], Stochastic Variational Inference (SVI) [4, 5], and Stochastic Gradient Langevin Dynamics (SGLD) [6, 7]).

A. Parameters for the tested methods

For ensemble-based methods (i.e., naive ensemble, batch ensemble, snapshot ensemble, and our proposed FastEnsemble), we trained six model checkpoints to estimate the uncertainty.

Naive ensemble We trained six models independently with different random seeds. The prediction results were generated by a simple average. The total computational budget is $6B$, where B is the budget to train one model from scratch.

FastEnsemble We first trained a standard checkpoint with budget B, then use $\frac{k_2+k_3}{k_1}B \times 5$ to obtain the rest 5 models. In total, it costed $\frac{k_1+5(k_2+k_3))}{k_1}B$. We chose $k_1 = 200$, $k_2 = k_3 = 6$ for the entire training task.

BatchEnsemble We replicated the batch ensemble code from official repository at https://github.com/google/edward2/blob/main/edward2/tensorflow/layers/convolutional.py#L560, and extended it to support ConvTranspose2d layer. We matched the training budget of our method by increasing the training time proportionally.

MC-Dropout We used the dropout rate equaling to $p = 0.5$. The computational budget is B.

SGLD We first trained the model until convergence (i.e., burn-in phase). At this stage, we did not inject Gaussian noise. During the inference time, we then trained the model for one epoch after each sampling where the learning rate was 1000x smaller than the training stage. No preconditioning technique was applied. We noted that although training budget was only B, the inference budget was much higher than other methods.

SVI We copied the implementation of MFVI from pyro (https://pyro.ai/examples/svi_part_i.html) and a 3rd-party implementation https://github.com/kumar-shridhar/PyTorch-BayesianDNN. The prior follows iid $\mathcal{N}(0, 0.02)$.

B. Quantification of the prediction accuracy

To quantify the performance of uncertainty assessment for individual methods, we used them to evaluate the impact of cell type mismatch and image impurities on prediction accuracy. To do this, we manually labeled the local regions that contain mismatched cells (LNCaP cells) or impurities using bounding boxes (red boxes in Fig. 6a). Here, the cell type mismatch dataset was created by artificially cutting images of LNCaP cells and superimposing them to images of MSCs. Examples of false positive (inside bounding boxes) and false negative (outside bounding boxes) predictions are denoted by yellow arrows in Fig. 6a).

We then defined the pixels inside and outside bounding boxes as positive $\mathcal{S}_1$ and negative $\mathcal{S}_2$ instances, respectively. These pixels were subsequently ranked by the uncertainty values in a descending order. The top-k highest uncertainty pixels $\mathcal{S}_2$ were selected. We then have TP@k $= |\mathcal{S}_1 \cap \mathcal{S}_2|$, Precision@$k$ = TP@k/k, and Recall@k = TP@$k|\mathcal{S}_1|$. Here, Precision@k and Recall@k report the model performance, and TP is the number of true positives.

Using the analyzed result, we then generated a receiver operating characteristic (ROC) curve with TP@k as the y-axis and FP@k as the x-axis (Fig. 6b and 6c) for each method. The ROC curve is a probability curve that reports the true positive rate (TPR) against false positive rate (FPR). To further quantify ROC curves, we computed the area under the curve (AUC) [8], in which

higher AUC values indicate better model performance in distinguishing the positive and negative classes. The results are summarized in Table S1.

We found that the ROC curves of the naive ensemble and our FastEnsemble method exhibit a very similar trend, while we note that the naive ensemble is *sim* 5× slower to train. The runner-up group is the snapshot ensemble and batch ensemble, which are as fast as our FastEnsemble method. Additionally, we found that traditional approximated Bayesian inference methods (i.e., SVI, SGLD and MC-Dropout), did not perform as well as other methods on the tested benchmark. Their suboptimal performance might be due to the approximation being too coarse to make compelling Bayesian inference, suggesting that a more precise Bayesian approximation is required for image translation applications.

2. FAST-ENSEMBLE TRAINING ALGORITHM

Algorithm S1. Algorithm of Fast-ensemble

Initialize: N: number of ensemble models parameterized by w_i; $\ell(\hat{y}, y)$: the loss function; λ: the hyperparameter to be tuned. $k_1 \gg k_2, k_3$: number of iterations for training seeding model, training sub-models and finetuning sub-models.
> Train the seeding model
for all $i \in \{0 \ldots k_1 - 1\}$ **do**
    Run one step of optimizer and learning rate scheduler.
Initial model list $\mathcal{M} = \{w_0\}$.
> Train the rest $N - 1$ models
for all $n \in \{1 \ldots N - 1\}$ **do**
    for all $i \in \{0 \ldots k_2 - 1\}$ **do**
        > Quick training
        One step update to minimize the augmented loss function in Eq. 2.
    for all $i \in \{0 \ldots k_3 - 1\}$ **do**
        One step update to minimize the standard loss function in Eq. 1. ▷ Finetuning
    Append to model list $\mathcal{M} = \mathcal{M} + w_n$.

3. ENSEMBLE MODEL OPTIMIZATION

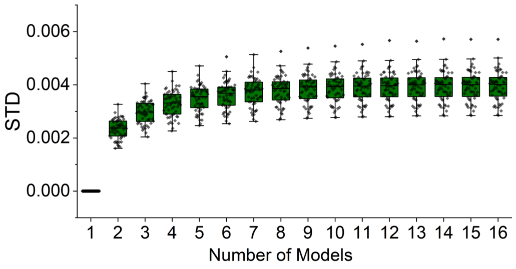

Fig. S1. Ensemble model optimization for model generation and performance. Six models achieved a sufficient STD result with the least computational load. Adding additional models added negligible improvement on statistics.

4. TRANSLATION OF PERTURBED IMAGES

To visualize the increased prediction uncertainty that arises from the image perturbation, we show the uncertainty maps of both perturbed and control (unaltered) images. As shown by the uncertainty maps in Fig. S2, all perturbed input images led to elevated uncertainty values for the cases of overexposure (Fig. S2a), non-uniform illumination (Fig. S2b), and wrong magnification (Fig. S2c). By calculating the target-prediction Pearson correlation coefficient, we confirmed that all imposed image perturbations lowered the AI prediction accuracy (Fig. S2d).

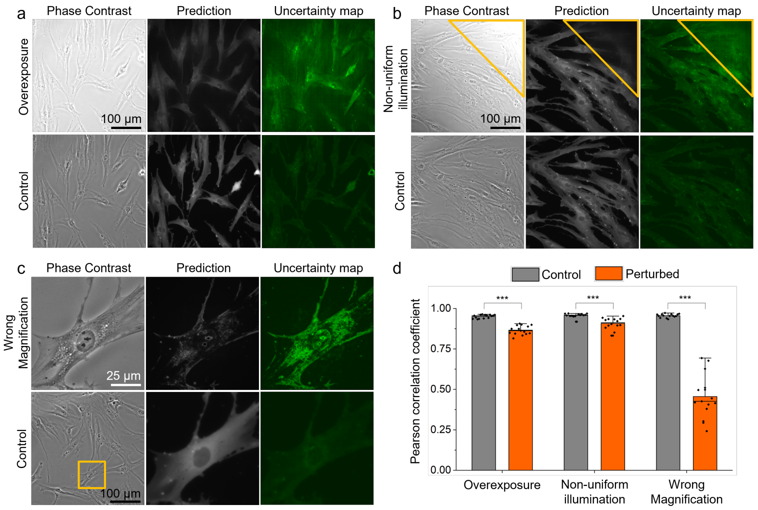

Fig. S2. Comparison of perturbed and corresponding control samples shown in Fig. 4 a-c Example phase contrast and prediction images showing influence of phase contrast perturbation on AI prediction results compared to unperturbed (Control) samples. For all control prediction images, a clear uniform distribution of CD105 can be reported. **d** Mean Pearson correlation coefficient was calculated per FOV for all conditions. We found that a significant drop in Pearson correlation coefficient can be observed for all perturbed FOVs compared to the corresponding control phase contrast image.

5. MODEL COMPARISON AUC RESULTS

Dataset	Naive	MC-Dropout	SGLD	SVI	BatchEnsemble	Snapshot	FastEnsemble
	Area under the ROC curve (AUC, controlling FPR$\leq$ 0.2)						
Image artifacts	**0.112**	0.074	0.099	0.050	0.098	0.002	0.108
Cell type mismatch	0.082	0.035	0.021	0.023	0.059	0.001	**0.090**
	Mean Pearson correlation						
20% Confluency	**0.974**	0.933	0.894	0.887	0.968	0.960	0.971
50% Confluency	**0.952**	0.909	0.853	0.849	0.947	0.939	0.950
80% Confluency	**0.925**	0.869	0.803	0.807	0.919	0.909	0.923
100% Confluency	**0.869**	0.803	0.756	0.762	0.865	0.853	0.865

Table S1. Experimental results in image generation benchmark. For clarity, the highest value is denoted in **bold**, second highest in red, and third highest in blue. Naive Ensemble performs best for all tested tasks except for one (cell type mismatch), confirming the superiority of ensemble methods over Bayesian inference methods in AI image translation tasks.

REFERENCES

1. Y. Wen, D. Tran, and J. Ba, "Batchensemble: an alternative approach to efficient ensemble and lifelong learning," arXiv preprint arXiv:2002.06715 (2020).
2. G. Huang, Y. Li, G. Pleiss, Z. Liu, J. E. Hopcroft, and K. Q. Weinberger, "Snapshot ensembles: Train 1, get m for free," arXiv preprint arXiv:1704.00109 (2017).
3. Y. Gal and Z. Ghahramani, "Dropout as a bayesian approximation: Representing model uncertainty in deep learning," in *international conference on machine learning*, (PMLR, 2016), pp. 1050–1059.
4. M. J. Wainwright and M. I. Jordan, "Introduction to variational methods for graphical models," Foundations Trends Mach. Learn. **1**, 1–103 (2008).
5. D. M. Blei, A. Kucukelbir, and J. D. McAuliffe, "Variational inference: A review for statisticians," J. Am. statistical Assoc. **112**, 859–877 (2017).
6. M. Welling and Y. W. Teh, "Bayesian learning via stochastic gradient langevin dynamics," in *Proceedings of the 28th international conference on machine learning (ICML-11)*, (Citeseer, 2011), pp. 681–688.
7. C. Li, C. Chen, D. Carlson, and L. Carin, "Preconditioned stochastic gradient langevin dynamics for deep neural networks," in *Thirtieth AAAI Conference on Artificial Intelligence*, (2016).
8. C. X. Ling, J. Huang, and H. Zhang, "Auc: A better measure than accuracy in comparing learning algorithms," in *Canadian Conference on AI*, (2003).
